# Rapidly Progressive Metastatic Adrenocortical Carcinoma With Oncocytic Features in a Young Male: A Case Report

**DOI:** 10.7759/cureus.91554

**Published:** 2025-09-03

**Authors:** Sultana Jannatun Nahar, G K M Rashik Uzzaman, Ibnul Shams, Muhammad Asif Iqbal Rao, Cornelius Fernandez

**Affiliations:** 1 General Medicine, United Lincolnshire Hospitals NHS Trust, Boston, GBR; 2 General Medicine, United Lincolnshire Hospitals NHS Trust, Lincolnshire, GBR; 3 Hematology, United Lincolnshire Hospitals NHS Trust, Boston, GBR; 4 Diabetes and Endocrinology, United Lincolnshire Hospitals NHS Trust, Boston, GBR; 5 Endocrinology, United Lincolnshire Hospitals NHS Trust, Lincoln, GBR

**Keywords:** adrenocortical carcinoma (acc), li-fraumeni syndrome, oncocytic variant, oncology, tp53 mutation

## Abstract

Adrenocortical carcinoma (ACC) is a rare but highly aggressive endocrine malignancy, often diagnosed at an advanced stage with limited treatment options and a poor prognosis. We present the case of a 29-year-old male who presented with pleuritic chest pain and was found to have a large right abdominal mass with widespread metastatic disease. Histological analysis revealed ACC with oncocytic features, a rare and distinct variant of ACC. Despite prompt diagnostic work-up and supportive care, the patient deteriorated rapidly. This case underscores the diagnostic challenges and dire prognosis associated with ACC and highlights the importance of early detection, multidisciplinary evaluation, and genetic testing with or without family history.

## Introduction

Adrenocortical carcinoma (ACC) is a rare malignancy of the adrenal cortex with an estimated annual incidence of 0.7-2.0 cases per million population [1]. Most patients present in the fifth to sixth decade of life, with a median age at diagnosis of 46-56 years. The disease typically manifests with symptoms of hormonal excess (Cushing’s syndrome and/or virilization) or nonspecific abdominal symptoms related to tumor mass effect [2]. The diagnosis of ACC is frequently delayed due to its insidious onset, the deep retroperitoneal location of the adrenal glands, and the heterogeneous nature that can complicate preoperative diagnosis [1,2].

## Case presentation

A 29-year-old previously healthy man presented with a four-week history of right-sided pleuritic chest pain, progressive dyspnea, anorexia, and an unintentional 15 kg weight loss. The patient was first referred to the chest clinic due to respiratory symptoms.

**Figure 1:**
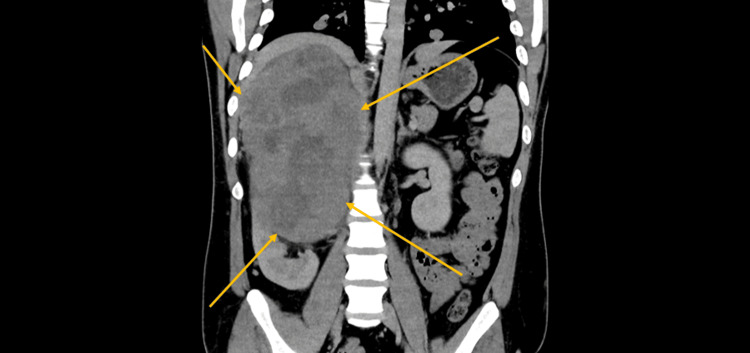
CT scan of the chest, abdomen, and pelvis revealed a 17 cm heterogeneously enhancing right-sided abdominal mass arising between the liver and right kidney, with internal necrosis, punctate calcifications, and invasion into adjacent retroperitoneal fat.

**Figure 2:**
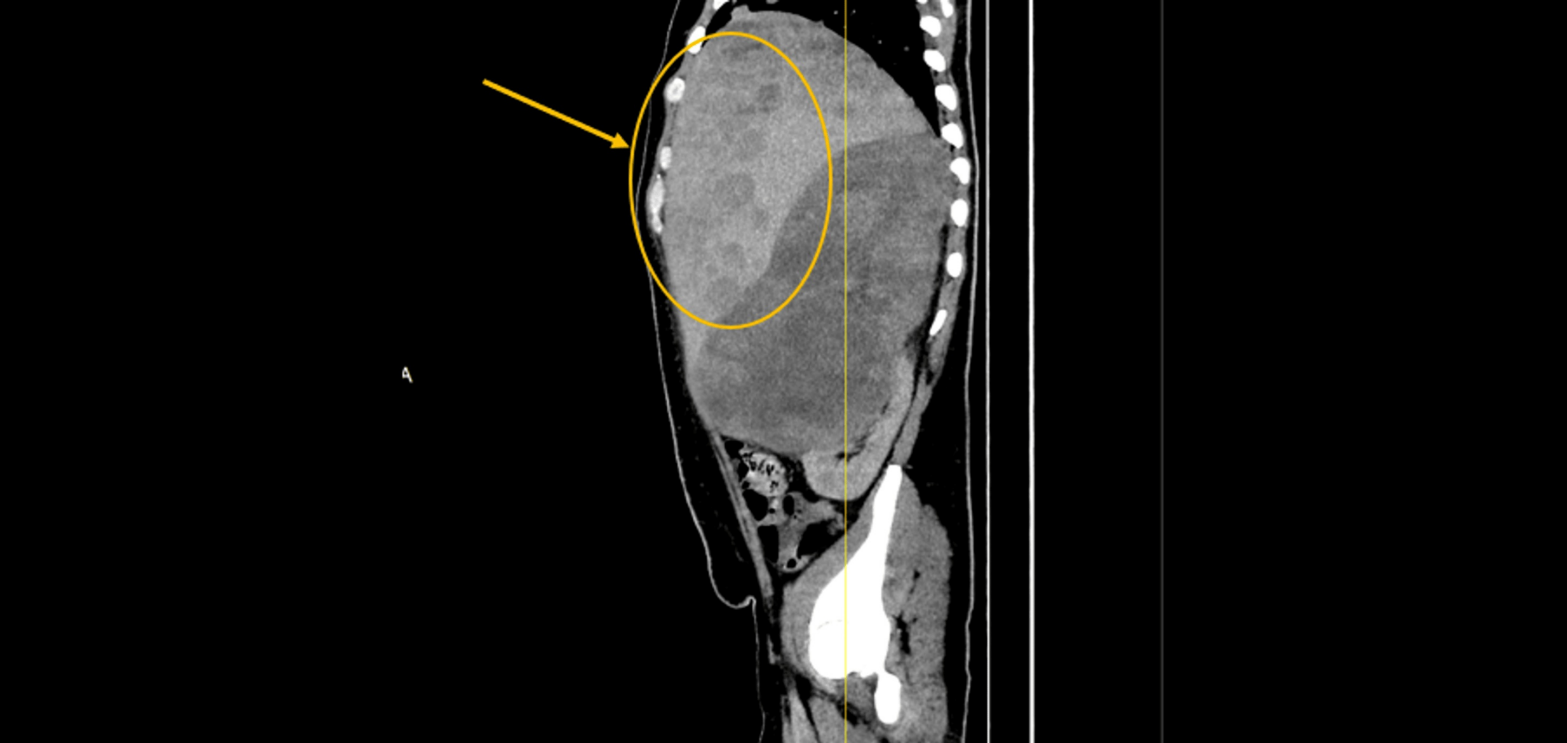
CT scan of the abdomen revealed multiple hypodense liver lesions, measuring up to 3.4 cm, which were consistent with hepatic metastases.

**Figure 3:**
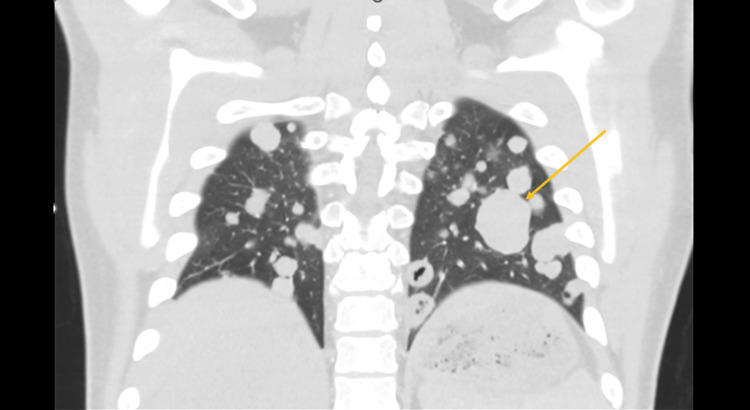
CT scan of the chest revealed bilateral pulmonary nodules, the largest measuring 1.9 cm in the right middle lobe, which suggested hematogenous dissemination.

Contrast-enhanced CT of the chest, abdomen, and pelvis revealed a 17 cm heterogeneously enhancing right-sided abdominal mass arising between the liver and right kidney, with internal necrosis, punctate calcifications, and invasion into adjacent retroperitoneal fat (Figure 1). Multiple hypodense liver lesions, measuring up to 3.4 cm, were consistent with hepatic metastases (Figure 2), and bilateral pulmonary nodules, the largest measuring 1.9 cm in the right middle lobe, suggested hematogenous dissemination (Figure 3). No significant mediastinal or retroperitoneal lymphadenopathy was identified.

**Figure 4:**
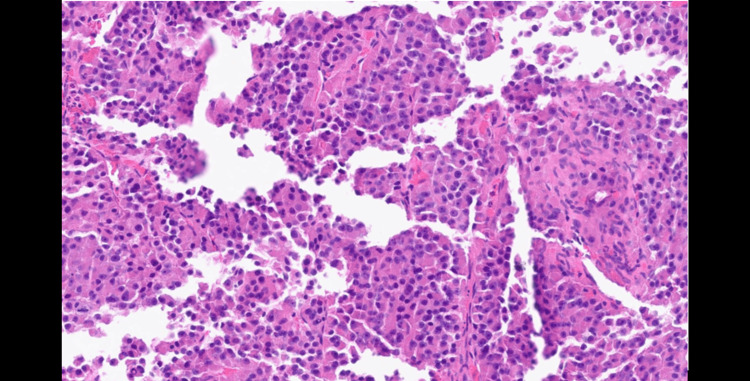
Biopsy of the abdominal mass revealed marked nuclear pleomorphism, prominent nucleoli, increased mitotic activity, and necrosis.

Subsequently, the patient was referred to the colorectal team, who organized a biopsy of the abdominal mass after an initial inconclusive liver biopsy. The US-guided Tru-Cut biopsy of the abdominal mass was suggestive of ACC. There was marked nuclear pleomorphism, prominent nucleoli, increased mitotic activity (16 mitoses per 50 HPF), necrosis, and vascular invasion (Figure 4). Immunohistochemical staining patterns showed positive Melan-A, Synaptophysin (focal), and S100; and negative AE1/3, Cam 5.2, and EMA. The Ki-67 index of >20% indicated a high proliferation rate. These findings were consistent with a high-grade oncocytic variant of ACC. The Lin-Weiss-Bisceglia (LWB) criteria for malignancy were met.

At this point, the patient was referred to the endocrine team. The case was promptly discussed in the adrenal MDT, which deemed the disease unresectable due to extensive local invasion and metastases; palliative chemotherapy was considered. The adrenal MDT also suggested genetic testing for TP53 mutation, which was negative, though the laboratory commented that this does not rule out Li-Fraumeni syndrome (LFS).

**Figure 5:**
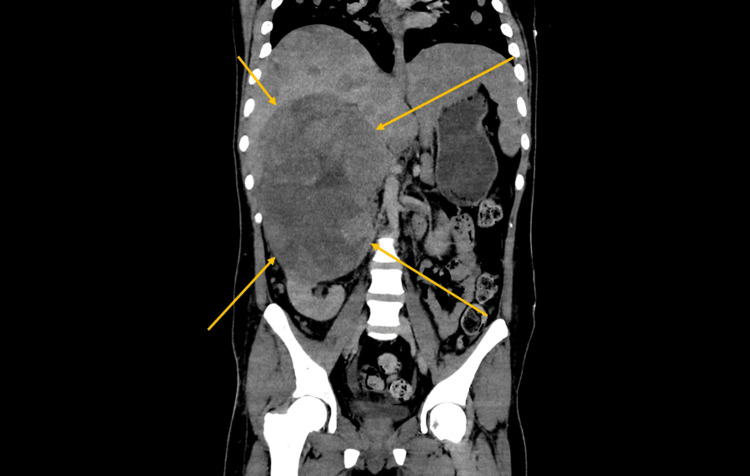
CT scan revealed progression in both size and metastatic spread of the adrenocortical cancer.

However, before initiation of palliative chemotherapy, the patient was admitted with sepsis, likely biliary in origin, and acute kidney injury. Repeat imaging demonstrated pneumobilia and progression of metastatic disease (Figure 5). He received broad-spectrum antibiotics, intravenous fluids, and low-molecular-weight heparin. Hormonal assessment was deferred due to sepsis; blood glucose remained normal. Despite maximal supportive measures, his condition deteriorated, and he subsequently died.

## Discussion

There are four subtypes of ACC with distinct features and prognoses, as summarized in Table 1. Oncocytic adrenocortical carcinoma (OACC) is an extremely rare histopathological variant characterized by tumor cells with abundant eosinophilic cytoplasm resulting from mitochondrial hyperplasia, with electron microscopy showing cytoplasm packed with numerous mitochondria. Fewer than 90 cases have been reported in the literature to date, with oncocytic cells constituting more than 90% of the tumor [3]. OACC poses significant diagnostic challenges due to overlapping morphological features with benign oncocytomas and other eosinophilic tumors. Unlike conventional ACC, OACC cannot be evaluated using the traditional Weiss scoring system because of the inherent characteristics of oncocytic cells [4].

**Table 1:** Subtypes of ACC with relative prevalence, median survival, and five-year survival. Source: Adapted from reference [5]. ACC, adrenocortical carcinoma

Subtype	Prevalence	Median survival	5-years survival
Conventional	~97%	Stage dependent I-24 y. IV=0.9 y	34-60% overall: <17% metastatic
Oncocytic	~2%	Benign: 41 mo; malignant 32.5 mo	70-93%
Myxoid	~0.8%	29 months	~29%
Sarcomatoid	~0.2%	6-7 months	<10%

Recent multicenter studies suggest that OACC demonstrates a more indolent clinical course with better overall survival and longer time to progression compared to conventional ACC [4]. We present a case of OACC that presented with abdominal symptoms related to the tumor mass effect, with no clinical features of hormonal excess as described in the literature; however, the patient had advanced disease at diagnosis with significant rapid progression, contrary to published literature.

The delayed diagnosis of ACC remains a significant clinical challenge, with the pre-existing adrenal mass being erroneously considered benign, the most common reason for diagnostic delay, occurring in 65% of cases. In up to 47% of cases, ACC may be misdiagnosed as other retroperitoneal malignancies [6]. The absence of reliable preoperative diagnostic markers compounds this challenge, as preoperative biopsy is contraindicated in suspected ACC due to the risk of tumor seeding. The non-specific symptoms in our case, including pleuritic chest pain and weight loss, which led to the discovery of an already advanced 17 cm mass with metastatic disease, illustrate the typical late presentation of ACC.

Non-functional ACC represents approximately 40% of all cases and typically presents with symptoms related to mass effect rather than hormonal excess. This is particularly relevant to the presented case, as the young male patient showed no clinical signs of hormonal excess such as Cushing’s syndrome or virilization. Non-functional tumors generally present later in the disease course compared to functional tumors, contributing to the poorer prognosis observed in this patient population [7]. The oncocytic variant specifically shows hormonal production in only 30-44% of cases, with most symptoms related to large tumor size and abdominal mass effects, including pain, discomfort, and weight loss [7]. This aligns with the presentation in this case, where the patient’s symptoms were primarily related to the mass effect.

In ACC, biochemical assessment of steroid hormone production is an essential diagnostic and prognostic step, as functional tumors may present with overt or subclinical hypercortisolism, virilization, or mineralocorticoid excess. Even in oncocytic variants, hormonal hypersecretion is reported in up to 30-44% of cases, making hormonal profiling a cornerstone of the initial work-up [7]. In our patient, hormonal assessment was delayed due to concurrent sepsis and critical illness, which limited immediate biochemical interpretation. While the final diagnosis confirmed a non-functional OACC, comprehensive hormonal testing would have provided additional diagnostic certainty and aligned with guideline-based practice.

The histological diagnosis of OACC presents unique challenges due to its distinctive morphological features. The LWB system is specifically recommended for evaluating OACC, as standard Weiss criteria may overestimate malignant potential in these variants. Major criteria for malignancy include a mitotic rate >5 per 50 high-power fields, atypical mitoses, or venous invasion, while minor criteria encompass large size (>10 cm and/or >200 g), necrosis, and capsular invasion. The presence of any major criterion indicates malignancy, while one to four minor criteria suggest uncertain malignant potential [8].

ACC arises sporadically in most patients but is linked to several hereditary cancer syndromes, as given in Table 2 [9]. Germline TP53 mutations in LFS confer high lifetime ACC risk, especially in children. Lynch syndrome (mismatch-repair gene defects) accounts for approximately 3% of adult ACCs, often with microsatellite instability. Beckwith-Wiedemann syndrome, marked by 11p15 imprinting alterations, predisposes infants to ACC. Carney complex, due to PRKAR1A mutations, carries multifocal endocrine tumors, including ACC. Rarely, MEN1 and familial adenomatous polyposis (APC) increase ACC susceptibility. Overall, approximately 10% of ACCs harbor germline mutations, underscoring the value of genetic testing for targeted surveillance and family counseling [10].

**Table 2:** Genetic syndrome associated with ACC. Source: Adapted from reference [10]. ACC, adrenocortical carcinoma

Syndrome	Causative gene(s)	Key features
Li-Fraumeni	TP53	Early-onset ACC: diverse cancers
Lynch	MLH1, MSH2, MSH6, PMS2	Microsatellite unstable tumors
Beckwith-Wiedemann	11p15 imprinting	Overgrowth, neonatal tumors
Carney complex	PRKAR1A	Pigmented lesions, endocrine tumors
MEN1	MEN1	Parathyroid, pituitary, adrenal
Familial adenomatous polyposis	APC	Colorectal polyps, extracolonic tumors

Genetic testing for ACC is strongly recommended in the UK, with all patients diagnosed with ACC being offered constitutional genomic testing for LFS regardless of age and for Lynch syndrome if under 50 years of age [9]. This recommendation reflects the established association between ACC and germline TP53 mutations, which can occur in any individual with ACC irrespective of age at diagnosis or family history. The UK Cancer Genetics Group has established comprehensive guidelines for TP53 testing, with ACC being one of the core LFS-related cancers. The UK surveillance protocol includes whole-body MRI and dedicated brain MRI from birth for confirmed TP53 carriers, with evidence suggesting a survival benefit from intensive surveillance.

Although the TP53 sequencing was negative, this does not definitively exclude LFS. Variant pathogenicity, incomplete coverage of sequencing methods, or mutations in other susceptibility genes (e.g., CHEK2, MDM2, CDKN2A) may still account for hereditary cancer predisposition [11]. Therefore, a negative TP53 test should be interpreted with caution, and genetic counseling with consideration of extended germline panel testing may still be warranted in selected patients.

Management of advanced or recurrent ACC remains challenging, with limited efficacy of conventional chemotherapy. In recent years, several therapeutic avenues have been explored. Targeted therapies directed against IGF-2/IGF-1R signaling, VEGF, and mTOR pathways have shown biological activity, although clinical benefit remains modest. More recently, immunotherapy has emerged as a promising strategy: checkpoint inhibitors such as pembrolizumab and nivolumab have demonstrated durable responses in small subsets of patients with mismatch-repair deficiency or high tumor mutational burden. Ongoing clinical trials are investigating combination strategies incorporating mitotane, immune checkpoint blockade, and targeted agents [12]. While these approaches are not yet standard of care, they represent important future directions for patients with aggressive or treatment-refractory ACC.

## Conclusions

This case underscores the critical importance of maintaining a high clinical suspicion for ACC in young patients presenting with large retroperitoneal masses, the urgent need for early detection strategies, and the need for genetic counseling and TP53 testing in all ACC patients to identify hereditary predisposition and guide family screening. The poor prognosis and limited treatment options for metastatic ACC highlight the pressing need for novel therapeutic approaches and emphasize the value of multidisciplinary care in optimizing patient outcomes.

## References

[1] Sharma E, Dahal S, Sharma P, Bhandari A, Gupta V, Amgai B, Dahal S (2018). The characteristics and trends in adrenocortical carcinoma: a United States population based study. J Clin Med Res.

[2] Else T, Kim AC, Sabolch A (2014). Adrenocortical carcinoma. Endocr Rev.

[3] Singh Y, Bharti JN, Chaoudhary GR (2022). Oncocytic adrenocortical carcinoma in a young patient. Acta Endocrinol (Buchar).

[4] Prinzi A, Guarnotta V, Di Dalmazi G (2025). Multicentric retrospective analysis of oncocytic adrenocortical carcinoma: insights into clinical and management strategies. Endocr Pathol.

[5] Minner S, Schreiner J, Saeger W (2021). Adrenal cancer: relevance of different grading systems and subtypes. Clin Transl Oncol.

[6] Ozsari L, Kutahyalioglu M, Elsayes KM (2016). Preexisting adrenal masses in patients with adrenocortical carcinoma: clinical and radiological factors contributing to delayed diagnosis. Endocrine.

[7] Scollo C, Russo M, Trovato MA (2016). Prognostic factors for adrenocortical carcinoma outcomes. Front Endocrinol (Lausanne).

[8] Urusova L, Porubayeva E, Pachuashvili N, Elfimova A, Beltsevich D, Mokrysheva N (2023). The new histological system for the diagnosis of adrenocortical cancer. Front Endocrinol (Lausanne).

[9] Hanson H, Brady AF, Crawford G (2020). UKCGG Consensus Group guidelines for the management of patients with constitutional TP53 pathogenic variants. J Med Genet.

[10] Else T (2012). Association of adrenocortical carcinoma with familial cancer susceptibility syndromes. Mol Cell Endocrinol.

[11] Ruijs MW, Broeks A, Menko FH (2009). The contribution of CHEK2 to the TP53-negative Li-Fraumeni phenotype. Hered Cancer Clin Pract.

[12] Kiesewetter B, Riss P, Scheuba C, Mazal P, Kretschmer-Chott E, Haug A, Raderer M (2021). Management of adrenocortical carcinoma: are we making progress?. Ther Adv Med Oncol.

